# Sparse-Scan Ptychography with Hybrid-Overlap Scanning and Reference-Guided Probe Updates

**DOI:** 10.3390/s26165168

**Published:** 2026-08-14

**Authors:** Sheng Chen, Zijian Xu, Ruoru Li, Xiangzhi Zhang, Renzhong Tai

**Affiliations:** 1 Shanghai Institute of Applied Physics, Chinese Academy of Sciences, Shanghai 201800, China; 2 Shanghai Synchrotron Radiation Facility, Shanghai Advanced Research Institute, Chinese Academy of Sciences, Shanghai 201204, China; 3 University of Chinese Academy of Sciences, Beijing 100049, China

**Keywords:** ptychography, sparse-scan, hybrid-overlap, ePIE

## Abstract

Sparse-scan ptychography accelerates data acquisition but compromises the overlap-induced redundancy essential for stable blind reconstruction. In low-overlap cases, object errors are often transferred to the probe update, causing crosstalk and artifacts. In this work, we propose a sparse-scan ptychography method termed hybrid-scan ptychography, which combines a hybrid-overlap scanning strategy with a reference-guided ePIE reconstruction algorithm. In this method, a localized high-overlap scan is used to establish a reliable probe estimate for reconstructing a wide field of view scanned on a low-overlap grid. As a stabilizing prior, this probe estimate is integrated into the ePIE probe update for the low-overlap region through an additional quadratic regularization term, thereby ensuring accurate probe reconstruction for sparse-scan region. This method effectively suppresses sparse-scan artifacts and ensures robust, high-fidelity reconstruction, as demonstrated by numerical simulations and soft X-ray ptychography experiments. With the new method, the acquisition efficiency of ptychography can be increased by at least 2 times without significant resolution reduction.

## 1. Introduction

Ptychography is a scanning coherent diffraction imaging technique in which a complex object and a probe function are simultaneously reconstructed from diffraction patterns acquired by overlapping scans [1,2,3,4]. Several key factors that determine practical ptychographic reconstruction quality have been clarified in previous works, including statistical noise treatment, scan sampling, and partial coherence [5,6,7]. Ptychography enables quantitative imaging with a spatial resolution surpassing the diffraction limits of conventional image-forming optics [8,9,10]. It has been applied in cryogenic biological imaging, spectroscopic, dichroic measurements, and Bragg crystallography [11,12,13,14,15] and has been implemented using electron and EUV [16,17,18,19]. These capabilities rely on the redundancy due to overlap between neighboring illuminated regions in ptychography, which links adjacent diffraction signals and constrains both object and probe recovery. Therefore, sufficient overlap is a practical requirement for stable ptychographic reconstruction.

However, the overlap requirement results in a substantial experimental burden. A high-overlap scan generally improves reconstruction convergence [3,6], but increases the number of diffraction patterns, scan time, and radiation dose on the sample. This burden becomes restrictive when a large field of view (FOV) must be covered, when the available coherent flux is limited, or when the specimen is sensitive to radiation damage. Sparse scanning reduces the number of exposure positions and is therefore desirable for dose- or time-limited measurements; however, it also weakens the redundancy that stabilizes simultaneous object–probe reconstruction.

Several sparse-scan reconstruction methods have been proposed to improve reconstruction quality from a sparse dataset. The alternating-direction method of multiplier (ADMM) solver and automatic-differentiation-based framework improve the optimization procedure and the consistency of data processing [20,21,22,23]. Total-variation (TV)-regularized and sparsity-promoting models introduce object-side prior information [24,25], and statistical formulations to account for noise in measurement. The performance of object-prior methods also depends on the compatibility of the selected prior with the specimen and on the chosen regularization strength. While these computational approaches could improve the quality of reconstruction, they inherently rely on the joint reconstruction of both the object and the probe from the same low-redundancy dataset. Consequently, they do not provide an independent physical constraint to prevent probe–object crosstalk when the scanning overlap is severely reduced.

In this work, we propose a hybrid-overlap scanning strategy and a reference-guided ePIE algorithm for sparse-scan ptychography, where a localized high-overlap reference scan is used to produce a reliable probe estimate. This estimate is integrated into the extended ptychographic iterative engine (ePIE) through a quadratic prior term, resulting in a new form of probe update for sparse region that preserves the algorithm’s sequential structure. The method, termed hybrid-scan ptychography, is evaluated with a Fresnel zone plate (FZP)-generated circular illumination and a Kirkpatrick–Baez (KB)-type square illumination in numerical simulations. The new method is also validated by a soft X-ray experiment on a standard sample.

## 2. Methods

### 2.1. Hybrid-Overlap Scanning and Reconstruction Workflow

During ePIE reconstruction, each measured diffraction pattern constrains the exit wave $\psi_{j}\left(r\right)$, rather than the object and probe separately. In early iterations, the object estimate is usually inaccurate and may contain unreliable information. The residual after the modulus projection therefore contains mixed contributions from errors in both the object and the probe. When this residual is used in the probe update, mismatch caused by an inaccurate object estimate may be partly redistributed into the probe, yielding an incorrect probe update. In a high-overlap scan, repeated measurements of the same object region under different adjacent illuminations constrain this object–probe separation and can correct the probe errors in later iterations. But in a low-overlap scan, these constraints from overlapping illuminated areas are much weaker, thus erroneous probe components from early iterations may persist and affect subsequent object updates. This can induce artifacts or even severe reconstruction degradation.

The above analysis indicates that, in the low-overlap scan, the sparse measurements alone do not provide sufficient constraints for robust probe updates. We therefore introduce a localized high-overlap reference scan within the sparse-scan region to recover a reliable probe estimate. This reference scan does not cover the full specimen; instead, it provides sufficient overlap for reconstructing a reference-probe. This reference probe serves as an additional constraint in the reconstruction of the wide-field sparse-scan data. As only the information of reconstructed probe, rather than the local object estimate, is transferred between the high-overlap loop and the low-overlap loop, the proposed method imposes no additional requirements on the specimen.

**Figure 1:**
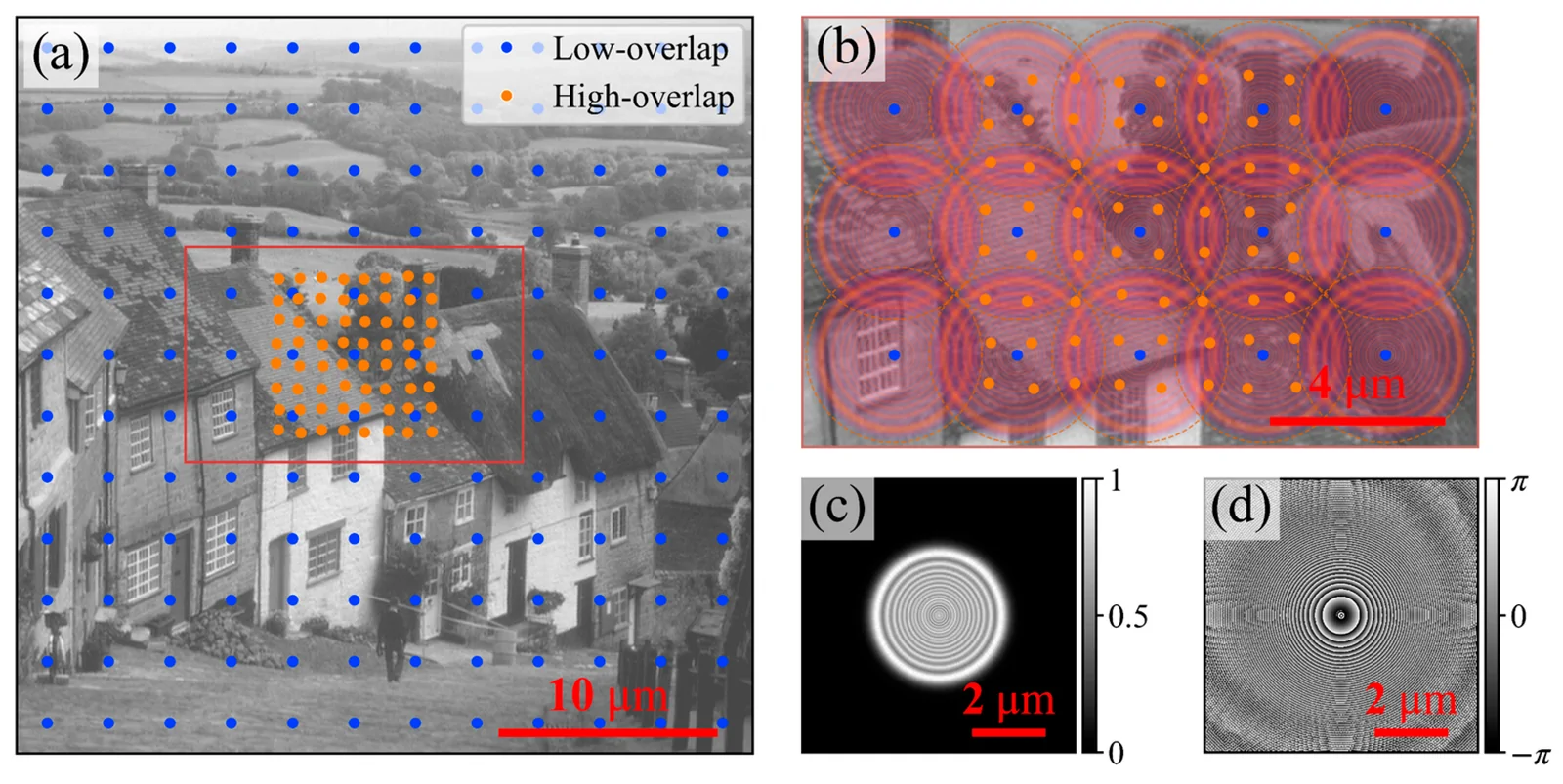
FZP simulation design. (**a**) Scan positions for the low-overlap step size of 2.8 µm. The blue and orange points denote low-overlap and high-overlap positions, respectively. (**b**) Enlarged red-boxed region of (**a**), where the red shaded circular regions are the footprints of the illumination spot. (**c**,**d**) Simulated probe intensity and phase at the sample plane, respectively.

The proposed acquisition strategy contains two regions on the sample, a high-overlap reference region and a low-overlap region (e.g., see Figure 1a), which are scanned in the same experimental run. The high-overlap reference region (dense region) is small but provides locally redundant diffraction data for reconstructing a high-quality probe. The low-overlap region (sparse region) covers the whole FOV (containing the high-overlap area) with a substantially reduced density of exposure positions, which is used to reconstruct the specimen. This hybrid-overlap design reduces the total number of scan positions while maintaining a strong local constraint for probe stabilization.

In the reconstruction, after an initial high-overlap warm-up stage, each complete iteration consists of a high-overlap probe-update loop and a low-overlap reference-guided update loop. In the high-overlap loop, a conventional ePIE algorithm is applied to the local high-overlap data, and the resulting probe estimate by this loop is denoted as a reference probe, $P^{ref}$. During the following low-overlap loop, the object is updated using the conventional ePIE algorithm, whereas the probe is updated assisted with $P^{ref}$ which is kept fixed in this loop. This assisted update is introduced below as the reference-guided update. Thus, $P^{ref}$ acts as an iteratively refreshed probe prior rather than as a one-time initial guess for the low-overlap region. It is used as an active regularization term in the probe update, thereby stabilizing the low-overlap region reconstruction.

### 2.2. Reference-Guided ePIE Method

Here we derive the probe-update formula used in the low-overlap reconstruction. The derivation is based on a local loss-function formulation of the ePIE probe update but augmented by a quadratic reference prior that penalizes deviation from the probe updated with the high-overlap reference region.

For the scan position $R_{j}$, the exit wave from the object is written as

(1) $$\psi_{j}\left(r\right)=P\left(r\right)O\left(r-R_{j}\right)\;\;\;\;\;\;\;\;$$

where $P\left(r\right)$ and $O\left(r\right)$ denote the probe and object functions, respectively. After a reciprocal-space modulus projection, the constraint-enforced exit wave is obtained as $\psi_{j}^{\prime }\left(r\right)$. The updated probe at this scan position is denoted as $P^{\prime }\left(r\right)$.

In conventional ePIE, the probe update can be implemented by minimizing a local loss function that combines a local data-consistency term together with a stabilizing term that keeps the updated probe close to the current estimate. To incorporate the reference probe $P^{ref}\left(r\right)$, we augment this loss function as

(2) $$D_{j}\left(P^{\prime }\right)=\sum_{r}{\left|P^{\prime }\left(r\right)O\left(r-R_{j}\right)-\psi_{j}^{\prime }\left(r\right)\right|}^{2}+\sum_{r}u\left(r\right){\left|P^{\prime }\left(r\right)-P\left(r\right)\right|}^{2}\;+\lambda_{j}\sum_{r}{\left|P^{\prime }\left(r\right)-P^{ref}\left(r\right)\right|}^{2}$$

where $u\left(r\right)$ is the ePIE stabilizing weight and $\lambda_{j}$ controls the strength of the quadratic reference prior for the *j*-th scan position. The first term enforces consistency with the constrained exit wave, the second term represents the standard ePIE proximal stabilization, and the third term introduces the reference-probe constraint.

To preserve the ePIE normalization, we define

(3) $$M_{j}=\underset{r}{max}{\left|O\left(r-R_{j}\right)\right|}^{2}$$

and choose

(4) $$u\left(r\right)=\frac{M_{j}}{\beta }-{\left|O\left(r-R_{j}\right)\right|}^{2}$$

where β is the probe relaxation parameter. Taking the Wirtinger derivative of $D_{j}$ with respect to the complex conjugate probe $P^{\prime *}\left(r\right)$ and setting it to zero, we have

(5) $$O^{*}\left(r-R_{j}\right)\left[P^{\prime }\left(r\right)O\left(r-R_{j}\right)-\psi_{j}^{\prime }\left(r\right)\right]+\left(\frac{M_{j}}{\beta }-{\left|O\left(r-R_{j}\right)\right|}^{2}\right)\left[P^{\prime }\left(r\right)-P\left(r\right)\right]\;+\lambda_{j}\left[P^{\prime }\left(r\right)-P^{ref}\left(r\right)\right]=0$$

Expanding Equation (5) and canceling the ${\left|O\left(r-R_{j}\right)\right|}^{2}P^{\prime }\left(r\right)$ terms yield the following

(6) $$\left(\frac{M_{j}}{\beta }+\lambda_{j}\right)P^{\prime }\left(r\right)=\frac{M_{j}}{\beta }P\left(r\right)-{\left|O\left(r-R_{j}\right)\right|}^{2}P\left(r\right)+O^{*}\left(r-R_{j}\right)\psi_{j}^{\prime }\left(r\right)+\lambda_{j}P^{ref}\left(r\right)$$

Using the current exit-wave estimate $\psi_{j}\left(r\right)=P\left(r\right)O\left(r-R_{j}\right)$, Equation (6) can be rewritten in form of increment as

(7) $$\left(\frac{M_{j}}{\beta }+\lambda_{j}\right)\left[P^{\prime }\left(r\right)-P\left(r\right)\right]=O^{*}\left(r-R_{j}\right)\left[\psi_{j}^{\prime }\left(r\right)-\psi_{j}\left(r\right)\right]+\lambda_{j}\left[P^{ref}\left(r\right)-P\left(r\right)\right]$$

For simplification, it is useful to express the reference-prior weight $\lambda_{j}$ as a dimensionless ratio with respect to the standard ePIE normalization,

(8) $$\lambda_{j}=\frac{\gamma M_{j}}{\beta }$$

where γ is defined as the reference-guiding strength parameter. Substituting Equation (8) into Equation (7) gives the closed-form reference-guided probe update,

(9) $$P^{\prime }\left(r\right)=P\left(r\right)+\frac{\beta }{\left(1+\gamma \right)M_{j}}O^{*}\left(r-R_{j}\right)\left[\psi_{j}^{\prime }\left(r\right)-\psi_{j}\left(r\right)\right]+\frac{\gamma }{1+\gamma }\left[P^{ref}\left(r\right)-P\left(r\right)\right]$$

When γ=0, Equation (9) reduces to the conventional ePIE probe update. For γ>0, the second term of the right side retains the data-driven ePIE correction with a reduced step size, while the third term provides a regularizing displacement toward $P^{ref}$. In the reconstructions below, γ=1 is used to balance the data-driven correction from the diffraction patterns and the regularization effect of the reference probe from the high-overlap scan region.

### 2.3. Reference-Guided Reconstruction Procedure

The complete reconstruction procedure of the proposed method is summarized in Algorithm 1. It is a combination of low-overlap reconstruction and high-overlap reconstruction. This combined procedure alternates between a high-overlap probe-update loop and a low-overlap loop in which the probe update is regularized by the refreshed reference probe.
**Algorithm 1:** Reference-guided reconstruction procedure with separated object estimates**Input:** high-overlap diffraction data $D_{H}$; low-overlap diffraction data $D_{L}$; initial probe $P^{\left(0\right)}$; initial object estimates $O_{H}^{\left(0\right)}$ and $O_{L}^{\left(0\right)}$; warmup iteration number $N_{warm}$; total iteration number $N_{iter}$
**Output:** separated high-overlap object $O_{H}$; low-overlap object $O_{L}$; reconstructed probe P. Set $O_{H}=O_{H}^{\left(0\right)}$, $O_{L}=O_{L}^{\left(0\right)}$, $P=P^{\left(0\right)}$
**for** iter in 1, …, $N_{warm}$ **do**        **for** each pattern *j* in $D_{H}$ **do**              Apply conventional ePIE updates to P and $O_{H}$ using pattern *j*              Keep $O_{L}$ as initialized        **end**
**end**
**for** iter in $N_{warm}$ + 1, …, $N_{iter}$ **do**        **for** each pattern *j* in $D_{H}$ **do**              Apply conventional ePIE updates to P and $O_{H}$ using pattern *j*        **end**        Assign $P^{ref}=P$ for the following low-overlap reconstruction loop        **for** each pattern *j* in $D_{L}$ **do**              Apply conventional ePIE object updates to $O_{L}$ using pattern *j*              Apply reference-guided ePIE probe update to P using $P^{ref}$        **end** **end** *Note:* The estimates $O_{H}$ and $O_{L}$ are separated for description purpose to independently evaluate the low-overlap reconstruction; the method itself does not require this object separation.

For evaluation purpose, the object estimate can be separated into a high-overlap object $O_{H}$ and a low-overlap object $O_{L}$. This separation is not required by the proposed reference-guided update. It is introduced only to isolate and independently evaluate the performance of the low-overlap reconstruction result, ensuring that the object estimate $O_{L}$ is not directly biased by updates from the high-overlap data. In a standard implementation, the reference-guided probe update can be applied to a common object estimate without being separated into two object estimates.

During the $N_{warm}$ warmup iterations, only the high-overlap data are used, and the conventional ePIE algorithm is applied to P and $O_{H}$, while $O_{L}$ is left unchanged. After the warmup iterations, each subsequent iteration contains two sequential loops. The high-overlap loop first visits all high-overlap patterns and applies conventional ePIE updates to $P^{ref}$ and $O_{H}$. The probe estimate obtained via this loop is then assigned to the initial probe of the low-overlap region and also used as the reference probe $P^{ref}$ for the following low-overlap loop, where $O_{L}$ is updated by the conventional ePIE algorithm and P is updated by the reference-guided probe update formula (Equation (9)) introduced in Section 2.2.

## 3. Results

### 3.1. Description of Simulation and Experiments

To evaluate the proposed hybrid-scan ptychography method which includes the hybrid-overlap scanning strategy and the reference-guided ePIE update algorithm described in Section 2, two simulations were conducted with different probe geometries: an FZP-generated circular probe and a KB-type quasi-square probe. The main simulation and experimental parameters are summarized in Table 1.

The simulated FZP case followed a defocused circular probe geometry, whereas the KB-type case used structured non-circular illumination. In both simulations, the high-overlap region was scanned with a jittered raster grid to reduce periodic artifacts in reconstruction [26], and the low-overlap region was scanned with a raster grid to achieve full and uniform coverage of the target FOV. The nominal linear overlap ratio is calculated as η=(D−s)/D, where D≈4 μm is the probe size and s is the low-overlap step size. The experimental dataset was acquired with an FZP illumination on a multi-Siemens-star specimen. The simulated reconstructions used a single-mode probe representation, and the experimental reconstructions used a five-mode probe representation. The reconstruction results mainly showed the low-overlap object estimate $O_{L}$ defined in Algorithm 1.

### 3.2. Simulation Results with FZP-Generated Probe

In the first simulation, an FZP with a diameter of 300 µm and an outermost zone width of 30 nm was used to produce the probe with an X-ray energy of 774 eV. The sample was placed 78 µm downstream of the FZP focus to obtain a 4 µm probe. The low-overlap scan used a 12 × 12 raster grid for the lowest overlap ratio (0.3) to cover an FOV of 30 µm × 30 µm, while the high-overlap scan used an 8 × 8 jittered raster grid with a 1.0 µm nominal step and ±0.1 µm positional jitter to cover a 7 µm × 7 µm area. For different low-overlap step sizes, the FOV kept unchanged. The largest-step hybrid dataset therefore contains 144 sparse-scan positions and 64 local dense positions (Figure 1). For comparison, a complete scan with the same 1.0 µm step size for the 30 × 30 µm FOV would require 31 × 31 = 961 positions.

All simulated reconstructions were run for 100 iterations with α = β = γ = 1.0, plus 10 warmup iterations for hybrid-scan reconstruction (i.e., $N_{iter}$ = 110). The diffraction data was noised with Poisson statistics to get a SNR of 20 ($SNR={\left\|I_{0}\right\|}_{2}/{\left\|I-I_{0}\right\|}_{2}$). For each low-overlap step size, two reconstructions were performed with different methods. The first reconstruction used the proposed reference-guided method (described in Section 2) and presented the separated low-overlap object estimate $O_{L}$. The second reconstruction used the conventional ePIE to process only the low-overlap data. This provided a baseline which isolates the effects of the local high-overlap reference probe and the guided probe update.

**Figure 2:**
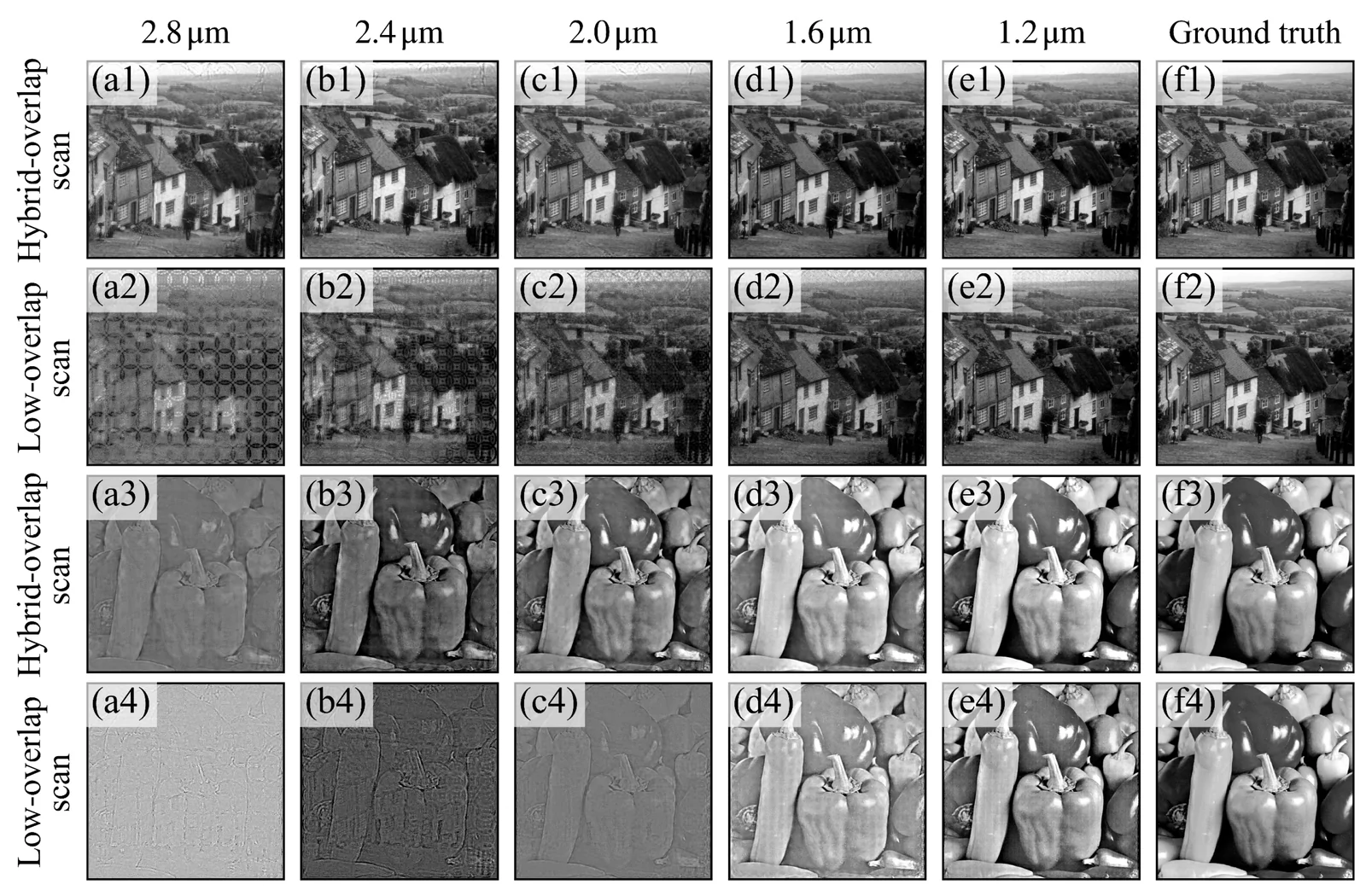
FZP simulation results. (**a1**–**e1**) Hybrid-overlap reconstructed amplitudes, (**a2**–**e2**) low-overlap-only reconstructed amplitudes, (**a3**–**e3**) hybrid-overlap reconstructed phase shifts, and (**a4**–**e4**) low-overlap-only reconstructed phase shifts. The four rows of panels (**a1**–**e4**) correspond to low-overlap step sizes of 2.8, 2.4, 2.0, 1.6, and 1.2 µm, respectively. (**f1**–**f4**) are the ground truth.

Figure 1 illustrates the FZP simulation geometry at the largest low-overlap step size (2.8 μm). As Figure 1a shows, the low-overlap positions cover a large FOV, whereas the high-overlap positions are confined to a localized region. The high-overlap region was chosen as a specimen-containing area to provide effective (penetrable) diffraction signals; subject to this condition, its location does not need to target any particular feature of the object. The enlarged view in Figure 1b shows that the largest step size places the sparse scan at a critical-coverage condition, where neighboring illuminated regions just critically cover the FOV without missing area. The simulated FZP probe intensity and phase (Figure 1c,d) at the sample plane were used to generate the diffraction data. The reconstructed object amplitude and phase for the hybrid-overlap and low-overlap-only schemes are presented in Figure 2.

As illustrated in Figure 2, the reconstruction quality of the low-overlap-only scan depends heavily on overlap ratio. At step sizes of 2.8 and 2.4 µm, the conventional ePIE reconstructions exhibit pronounced grid-like artifacts in the amplitude images, and the phase images barely show object features. These artifacts should be associated with insufficient separation between the object and probe functions from the exit wave. When the redundancy provided by overlap is limited, the object-related mismatch at early iterations may be partly absorbed by the probe update rather than corrected through neighboring overlap constraints.

With the hybrid-scan ptychographic reconstruction, these artifacts were greatly reduced by the reference-guided ePIE updates. Residual artifacts were still visible in the sparsest scan case of 2.8 µm step size, but the global morphology was mostly recovered, and the amplitude image was even better reconstructed compared to the conventional ePIE low-overlap result. At the intermediate step size of 2.0 µm, the hybrid-scan reconstruction was barely affected by artifacts, whereas the low-overlap reconstruction showed only partial recovery of the underlying object structures.

As the step size further decreases to 1.6 and 1.2 µm, the discrepancy between the two strategies gradually diminishes. This convergence in quality suggests that the inherent redundancy of data eventually becomes sufficient for stable blind reconstruction. These results indicate a useful mechanism that the low-redundancy data is insufficient for probe recovery but still retain sufficient inter-position constraints for object reconstruction.

### 3.3. Simulation Results with a KB-Type Quasi-Square Probe

**Figure 3:**
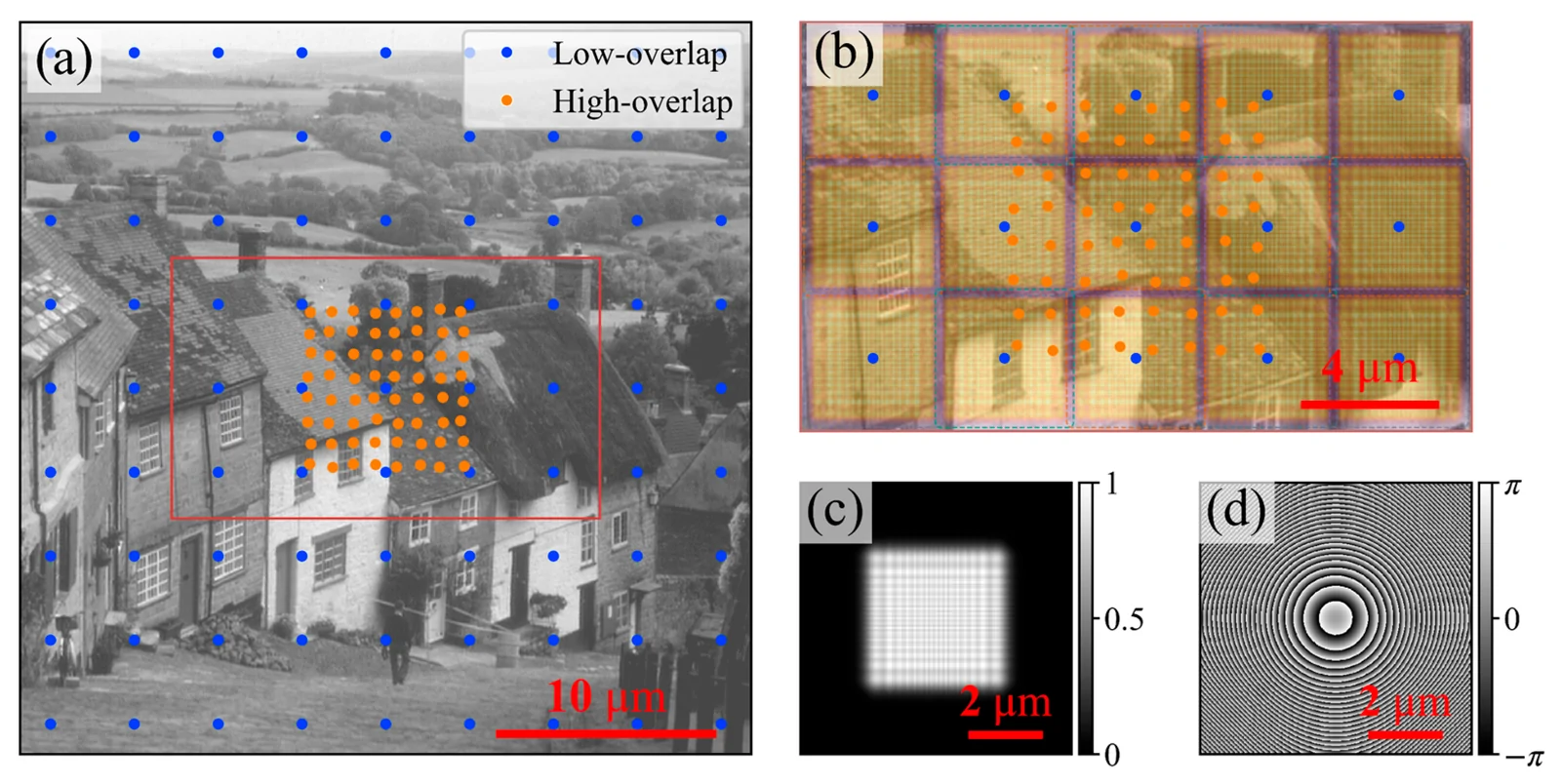
KB-type probe simulation design. (**a**) Scan positions for the low-overlap step size of 3.8 µm; the blue and orange points denote the low-overlap and high-overlap positions, respectively. (**b**) Enlarged red-boxed region of (**a**), where the yellow shaded square regions are the footprints of the square illumination spot. (**c**,**d**) Simulated probe intensity and phase, respectively.

To further evaluate the proposed strategy for different illumination geometries, the same simulation scheme was applied to the case of a KB-type quasi-square probe, which was generated with an ideal focusing model from a 10th-order super-Gaussian pupil of 500 µm width. In this case, the high-overlap region was scanned on an 8 × 8 grid, which was also used for the sparsest low-overlap scan. The imaged FOV, jitter amplitude, initialization, noise level, iteration number, and reconstruction parameters were the same as those in the FZP simulation. The low-overlap step size series were 3.8, 3.2, 2.6, 2.0, and 1.4 µm. These step sizes were chosen according to the shape and size of the illumination spot rather than directly copying from the FZP step sizes. The KB-type quasi-square probe and its hybrid scanning setup are shown in Figure 3.

**Figure 4:**
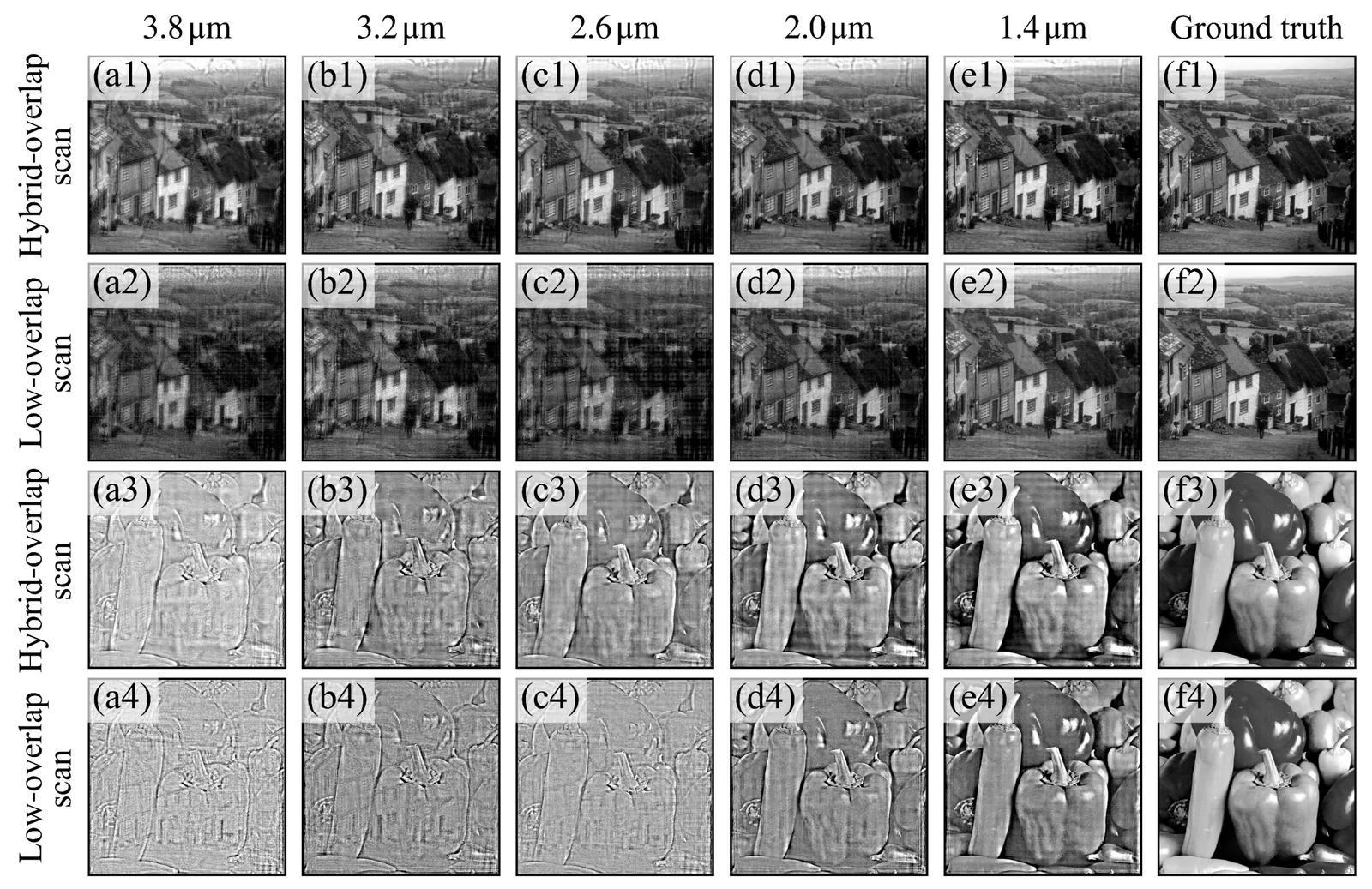
KB-type simulation results. (**a1**–**e1**) Hybrid-overlap reconstructed amplitudes, (**a2**–**e2**) low-overlap-only reconstructed amplitudes, (**a3**–**e3**) hybrid-overlap reconstructed phase shifts, and (**a4**–**e4**) low-overlap-only reconstructed phase shifts. The four rows of panels (**a1**–**e4**) correspond to low-overlap step sizes of 3.8, 3.2, 2.6, 2.0, and 1.4 µm, respectively. (**f1**–**f4**) are the ground truth.

The simulated reconstruction results for the KB-type probe case are shown in Figure 4. The trend in Figure 4 is similar to that in the FZP-probe simulation. As the sparse step size is increased, the hybrid-scan reconstructed image is much less degraded than the low-overlap-only image which contains strong artifacts, although residual distortions remain in the hybrid-scan results for the largest step sizes. The comparison between Figure 4 and Figure 2 highlights that the impact of low-overlap step size should be evaluated in conjunction with the illumination geometry and the related overlap redundancy.

For the simulated reconstructions, the object quality can be quantified using a gauge-corrected normalized root-mean-square error (NRMSE) [22]. Because ptychographic reconstruction contains a global complex scaling ambiguity, the reconstructed object is first aligned to the ground truth by a complex scalar, then its relative deviation from the ground truth is calculated as

(10) $$NRMSE_{O}=\frac{\underset{a\in C}{\min}{\left\|aO-O_{true}\right\|}_{2}}{{\left\|O_{true}\right\|}_{2}}$$

where O is the reconstructed object and $O_{true}$ is the ground truth. The minimization over a removes the global gain and phase ambiguity before the reconstruction error is evaluated. The factor a is computed as

(11) $$a=\frac{\sum_{r}O^{*}\left(r\right)O_{true}\left(r\right)}{\sum_{r}{\left|O\left(r\right)\right|}^{2}}$$

**Figure 5:**
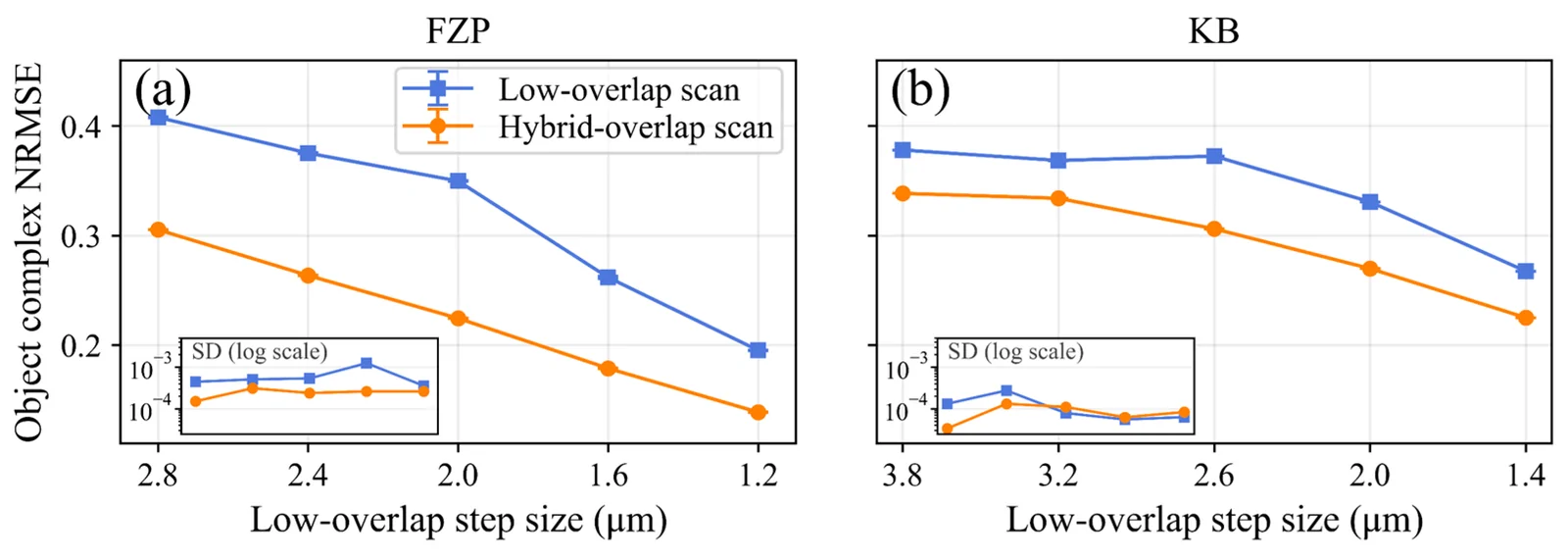
Reconstructed object NRMSE for the simulated reconstructions with five independent reconstruction runs for each case. (**a**) Object errors for the FZP circular probe case. (**b**) Object errors for the KB-type square probe case. NRMSE was calculated after global complex alignment to the ground truth. Markers and error bars denote the mean and ±1 standard deviation (SD) values of the five reconstructions, respectively; the insets show the corresponding SDs on a logarithmic scale.

The quantitative comparison in Figure 5 is consistent with the reconstruction image series in Figure 2 and Figure 4. For both probe geometries, the mean object NRMSE shows an overall decrease as the low-overlap step size is reduced, and the hybrid-scan results consistently maintain a lower mean error than the low-overlap-only results across the entire tested range.

The simulation results presented here utilized regular raster scans for low-overlap cases to achieve uniform coverage. In addition, the simulations of hybrid-overlap scanning on low-overlap jittered raster grids have also been performed and their results showed the same overall trend (Supplementary Figures S1–S3). All these results demonstrate that the proposed method consistently improves reconstruction quality regardless of the specific scanning trajectory.

### 3.4. Experimental Results

An experimental test was performed at the BL08U1A beamline of Shanghai Synchrotron Radiation Facility. In this experiment, a multi-Siemens-star specimen was scanned by an annular FZP illumination that was generated by moving the sample downstream of the FZP focus. The FZP used here has the same parameters as the modeled one in Section 3.2. The low-overlap step sizes were selected as 2.0, 1.9, 1.8, and 1.7 µm, and the high-overlap step size was 1.0 µm. The reconstruction for each sparse step size was performed with 1000 iterations for hybrid reconstruction plus 60 warmup iterations (i.e., $N_{iter}$ = 1060), while the remaining parameters were kept consistent with those in Section 3.2. The specific experimental setup is summarized in Table 1. Compared to simulations, a much larger total iteration number is used here.

In detail, the sample was positioned 75 μm downstream of the FZP focus, producing an annular illumination of approximately 4 μm in diameter at the sample plane. The diffraction patterns were recorded by a detector with an 11 μm pixel pitch at a sample-to-detector distance of 95.6 mm. At 774 eV, this geometry provided a coverage of radial momentum transfer up to $0.423\;{nm}^{-1}$. As shown in Supplementary Figure S4, the azimuthally averaged diffraction intensity yielded a critical momentum transfer of approximately $0.20\;{nm}^{-1}$, marking the upper boundary of the effective momentum-transfer range.

**Figure 6:**
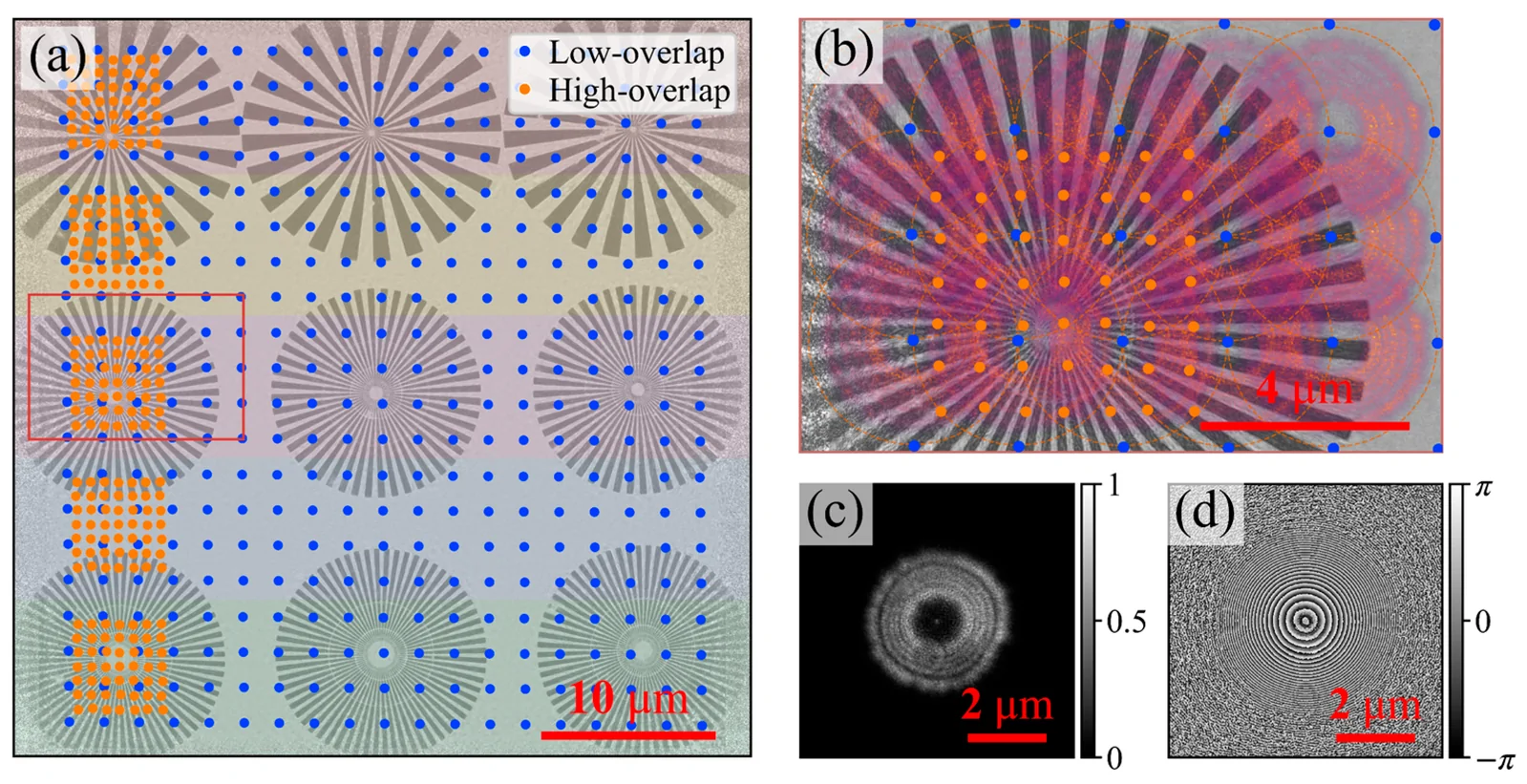
Experimental scan design and reconstructed probe. (**a**) Scan positions for the largest low-overlap step size of 2 µm; the blue and orange points denote the low-overlap and high-overlap positions, respectively. The colored backgrounds indicate five partitions of the FOV, and each partition has an independent probe. (**b**) Enlarged red-boxed region in (**a**). (**c**,**d**) Final reconstructed probe intensity and phase, respectively, from one of the partitions.

In this experiment, the maximum low-overlap step was limited to 2.0 µm because of the annular FZP probe structure. Due to the central stop of the FZP, the defocused probe on the specimen has a low-intensity ‘hole’ at the center which must be covered by the high-intensity rings of neighboring illuminations to ensure continuous data coverage. This requirement restricts the low-overlap spacing to approximately the probe radius, precluding sparser scanning. The experiment was therefore intended to demonstrate the practical implementation of the proposed workflow under a realistic annular soft-X-ray illumination. Figure 6 shows the scan geometry in experiments and a reconstructed probe. The low-overlap raster grid covers the larger specimen area, while the five high-overlap scan areas are much smaller. The reconstructed probe contains structured amplitude and phase features, as expected for an annular FZP illumination with a central hole.

In the experimental reconstruction, a five-mode probe representation was used under the state-mixture algorithmic framework [7]. Also, the iterative partition reconstruction method was used to reduce the influence of position-dependent probe variations over the large scan area [27]. In this method, the scan positions were divided into several spatial partitions, as exemplified by the colored backgrounds in Figure 6a. Each partition had its own probe estimate and its own reference probe $P^{ref}$, and the reference-guided update in Equation (9) was applied into each partition’s inner loop using the partition-specific reference probe. The iterative partition reconstruction method used here is to account for position-dependent probe instability within the proposed reference-guiding reconstruction framework, rather than an additional reconstruction principle investigated in this work.

**Figure 7:**
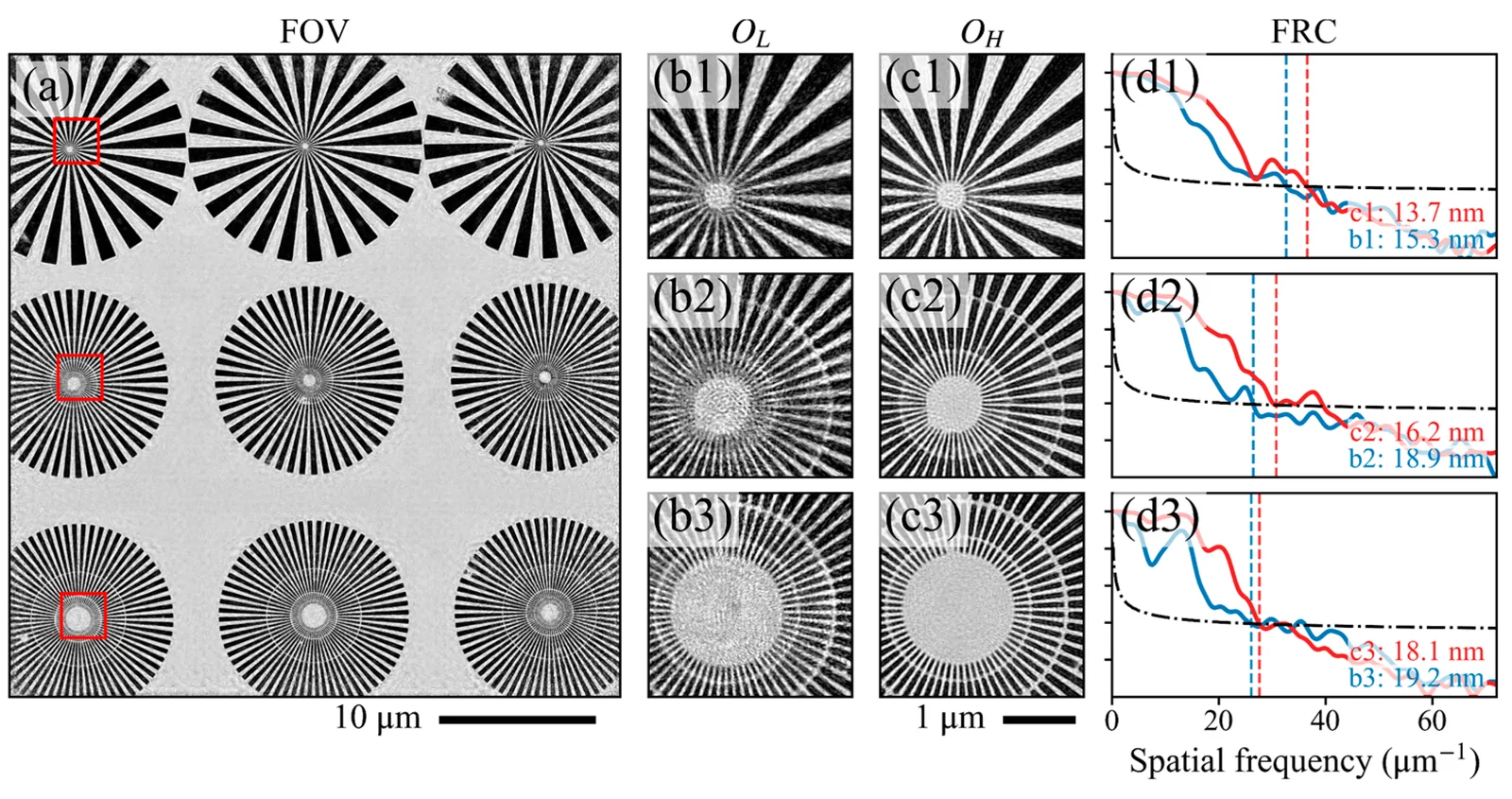
Experimental reconstruction results for the low-overlap step size of 2.0 µm with separated object updates. (**a**) Entire reconstructed FOV with selected ROIs marked by red boxes. (**b1**–**b3**) Enlarged ROIs from the low-overlap object reconstruction $O_{L}$. (**c1**–**c3**) The corresponding areas from the high-overlap object reconstruction $O_{H}$. (**d1**–**d3**) The FRC curves computed within the corresponding ROIs, annotated with resolution estimates that are indicated by the blue and red dashed lines for $O_{L}$ and $O_{H}$, respectively.

The reconstruction result of the experimental data for the low-overlap step size of 2.0 µm is shown in Figure 7, where $O_{L}$ and $O_{H}$ are compared in detail. $O_{H}$ should be regarded as an internal high-overlap reference reconstruction rather than an experimental ground truth. This comparison aims to evaluate the efficacy of the proposed hybrid-overlap scanning strategy and the reference-guided update algorithm in facilitating sparse region reconstruction. Specifically, it examines whether the low-overlap reconstruction $O_{L}$, when constrained by the reference probe, can maintain morphological fidelity comparable to the high-overlap reference $O_{H}$, without direct data sharing between the two object functions during the object updates.

Through observation on the regions of interest (ROIs) chosen in Figure 7a and enlarged in Figure 7(b1–c3), we can see that the spokes and most structure boundaries are preserved well in $O_{L}$, although the local contrast and background smoothness of $O_{L}$ are inferior to that of $O_{H}$. These behaviors are consistent with the presumed role of the reference-guided update: it improves the visibility of the low-overlap reconstruction through the probe constraint, although the low-overlap reconstruction quality still falls short of the high-overlap case. In addition, the spatial resolution is estimated for each ROI using Fourier ring correlation (FRC) analysis [28], and the related curves are shown in Figure 7(d1–d3). For all the three selected ROIs, the half-period spatial resolutions are evaluated under the 1-bit threshold criterion, demonstrating that the low-overlap reconstruction yields spatial resolutions closely matching those of the high-overlap reference reconstructions.

**Figure 8:**
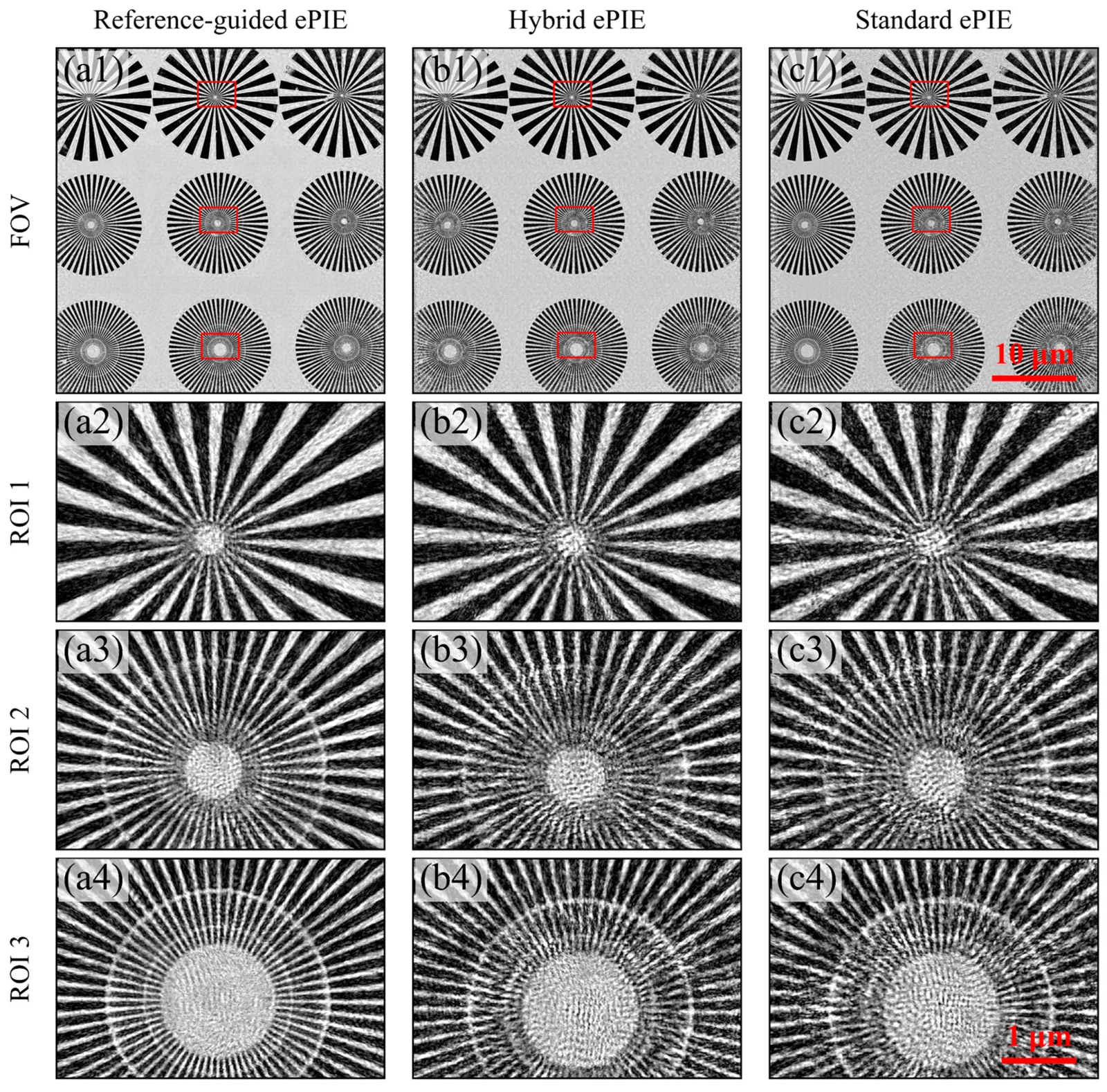
Comparison of experimental reconstructions using three approaches for the 2.0 µm step size dataset. The reconstructed amplitudes of low-overlap object $O_{L}$ are shown here. The first row of panels (**a1**–**c1**) show the whole reconstructed specimen using the proposed reference-guided ePIE, hybrid ePIE, and standard ePIE, respectively. The three columns of panels (**a2**–**c4**) are enlarged images of the corresponding ROIs in (**a1**–**c1**), respectively. These ROIs are marked by red boxes in (**a1**–**c1**).

Improvements of the proposed reconstruction method compared to conventional ePIE-based approaches are shown in Figure 8. Here, two control reconstruction schemes were carried out using the experimental dataset of 2.0 µm sparse step size. In the first control case, the reference-guided probe update was replaced with a conventional ePIE probe update in the low-overlap loop of Algorithm 1. This case is referred to as hybrid ePIE. In the second case (denoted as standard ePIE), a standard ePIE workflow was used to reconstruct the dataset without distinguishing between high- and low-overlap regions. The reconstruction using the proposed method is denoted as reference-guided ePIE. Because the regularization parameter γ=1.0 in Equation (9) effectively reduces the probe update strength by half, we set the probe update step size to β=0.5 for both control cases to maintain parity.

The comparison in Figure 8 demonstrates that the proposed method (reference-guided ePIE) recovers finer structural features and significantly enhances reconstruction fidelity compared to the other two conventional approaches. The benefits are clearly observable in the third row of panels, where the reference-guided ePIE reconstruction shows a cleaner background and lower noise level compared to the controls. Furthermore, the magnified views in the fourth row highlight that the proposed method resolves the finest spokes of the Siemens star, whereas the control reconstructions exhibit blurring and artifacts that degrade the resolutions of these images.

**Figure 9:**
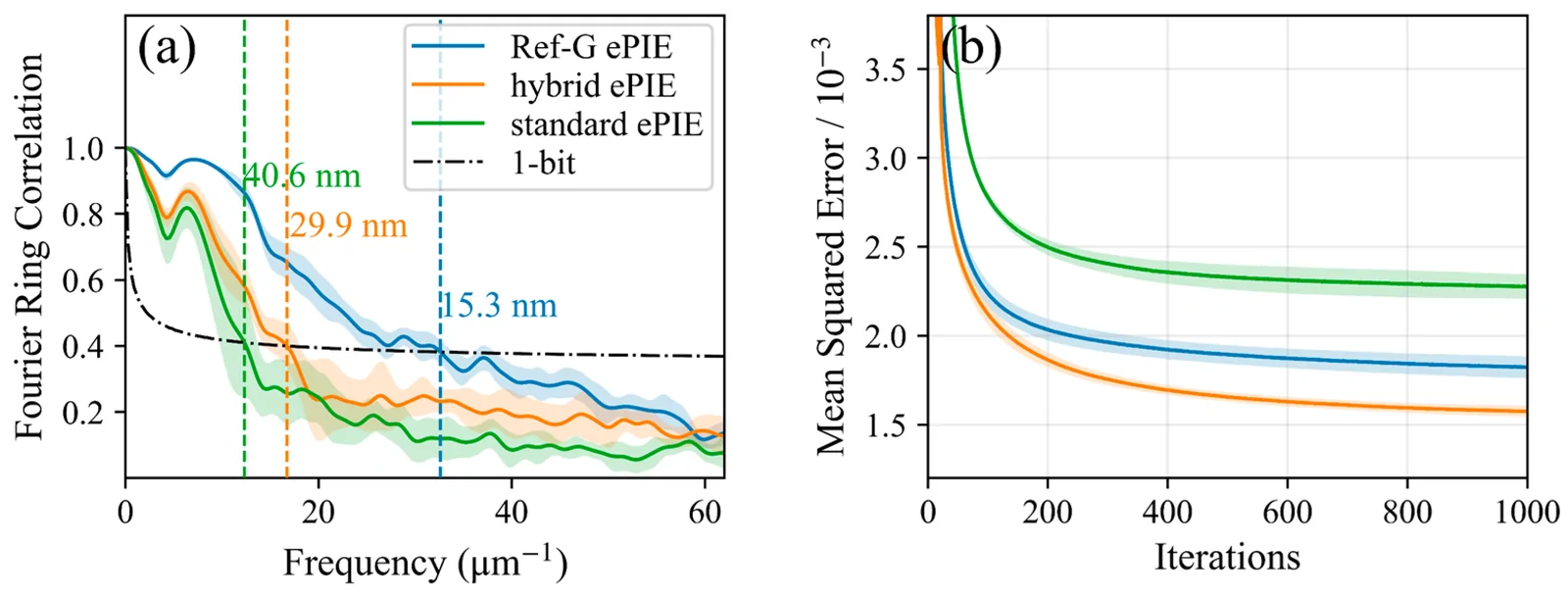
FRC and MSE analyses of the three reconstructions using different approaches. (**a**) FRC curves for the low-overlap regions reconstructed by the proposed approach (denoted as Ref-G ePIE), hybrid ePIE and standard ePIE, respectively, with the 1-bit threshold curve shown together. (**b**) Normalized far-field amplitude MSE curves for the three reconstructions. Solid curves and shaded bands denote the mean and ±1 SD values over five independent reconstruction runs, respectively.

Figure 9 summarizes the resolution estimates and convergence behavior of the experimental reconstructions using the three approaches. The spatial resolution for each reconstructed image is evaluated using FRC analysis. In both panels, the solid curve and shaded band denote the mean and ±1 SD over five independent reconstructions, respectively; the resolutions are obtained from the mean FRC curves in Figure 9a. As shown in Figure 9a, the object reconstructed by the proposed approach achieves a half-period spatial resolution of 15.3 nm under the 1-bit threshold criterion, which is much higher than the other two results.

The mean-square error (MSE) was used to characterize the data-consistency convergence behavior during iterative reconstruction. It is calculated from the normalized far-field amplitude mismatch:

(12) $$MSE=\frac{\sum_{j}{\left(\;\sum_{k}\left|\left|\Psi_{j}\left(k\right)\right|-\sqrt{I_{j}\left(k\right)}\right|\right)}^{2}}{\sum_{j}\sum_{k}I_{j}\left(k\right)}$$

where $\Psi_{j}\left(k\right)$ is the calculated far-field diffractive wave at scan position *j* and $I_{j}\left(k\right)$ is the measured diffraction intensity. For Figure 9b, the warm-up iterations were excluded, and all diffraction data were used for the reconstruction starting from iteration 0. As shown in Figure 9b, all the three MSE curves decrease with iteration number and eventually stabilize. Finally, the hybrid ePIE reconstruction reaches a slightly lower MSE than the reference-guided ePIE reconstruction. This behavior is related to the principle of the reference-guided update: by preventing the probe from erroneously absorbing residual during reconstruction, the reference-guided workflow leaves part of the noise-induced mismatch in the final data-consistency error rather than fitting it into unstable probe corrections.

**Figure 10:**
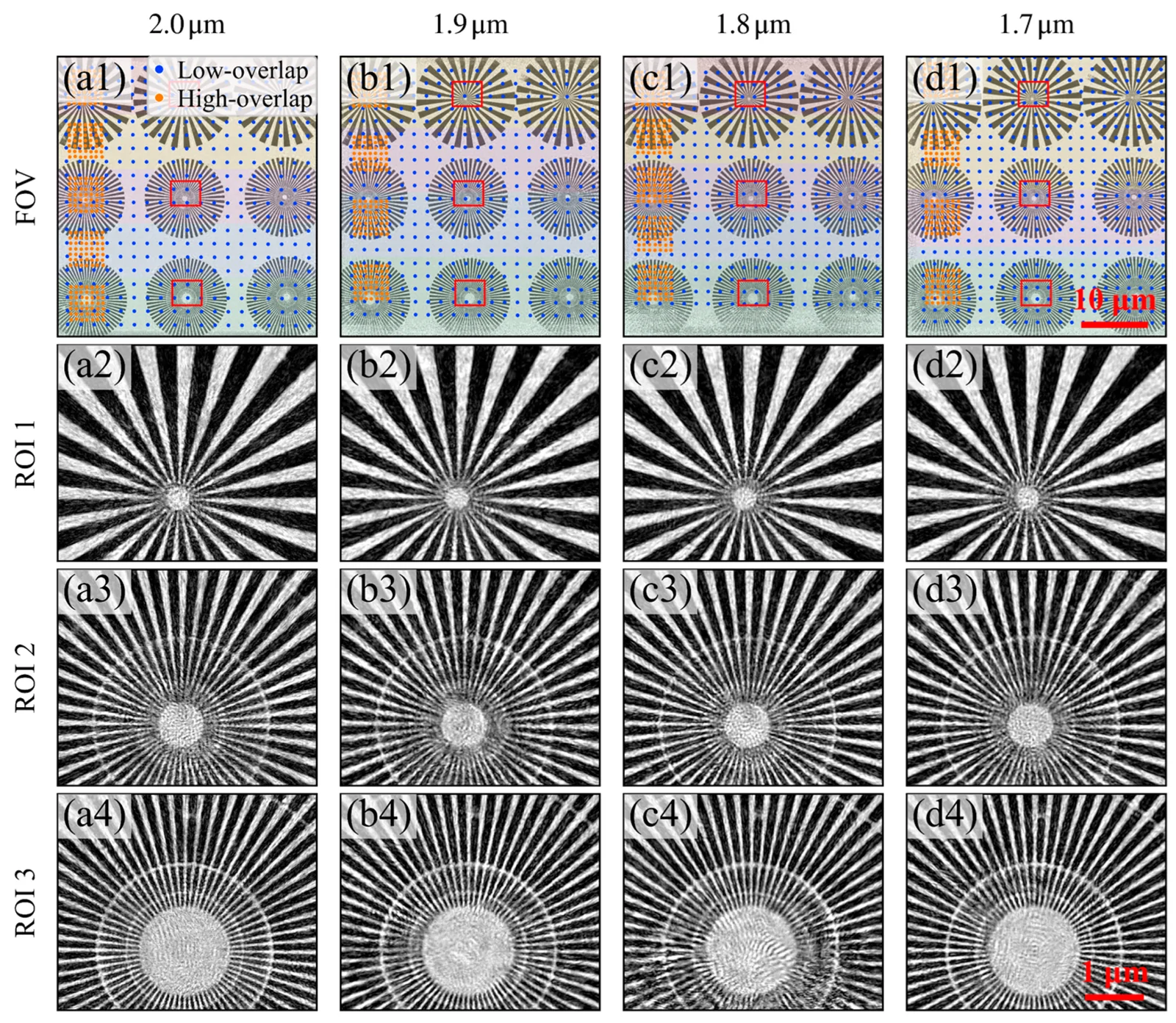
Experimental reconstruction results of the low-overlap object $O_{L}$ for the low-overlap step sizes of 2.0, 1.9, 1.8, and 1.7 µm, respectively, with the partitioned layout indicated in the first row using different colors. The first row of panels (**a1**–**d1**) show the whole reconstructed specimen with the four step sizes, respectively. The red boxes in (**a1**–**d1**) indicate selected ROIs. The four columns of panels (**a2**–**d4**) show enlarged images of the corresponding ROIs in (**a1**–**d1**), respectively.

Figure 10 gathers the $O_{L}$ reconstructions for the four sparse step sizes. The overall Siemens-star structures are visible across the tested FOV, but the local image quality depends not only on the nominal step size but also how each ROI is covered by the annular illuminations. The scan-position maps in the top row are therefore important for interpreting the local images. For example, in the case of 1.8 µm step size, Figure 10(c1) shows that the lower part of ROI 3 is not fully covered by the near annular illuminations, resulting in significant degradation of image quality in the lower part of Figure 10(c4). The resolution estimates and convergence behavior are provided in the Supplementary Figure S5, containing FRC curves and MSE curves, which indicate that all the four low-overlap reconstructions converged and achieved spatial resolutions of about 20 nm or even better under the 1-bit threshold criterion.

In addition, the proposed reconstruction algorithm primarily stabilizes the probe evolution through the reference-guided term and thereby reduces the influence of illumination-reconstruction errors. It does not explicitly correct scan-position errors, inaccuracy of sample–detector distance, weak sample and probe fluctuations, or other uncertainties during acquisition. In conventional high-overlap experiments, these non-ideal factors are often partially suppressed by high-overlap redundancy. In the present low-overlap reconstruction, the elimination of these factors’ influence depends more on experimental stability, model accuracy, and local illumination coverage condition.

## 4. Discussion

Both the simulation and the multi-Siemens-star experiment results indicate that the proposed hybrid-scan method can reduce the low-overlap reconstruction degradation by introducing a localized high-overlap scan for probe recovery and using the high-overlap probe as a guiding term in the low-overlap update. As a result, the new strategy can increase the acquisition efficiency of ptychography by at least two times without significantly decreasing resolution.

Although the update equation in Section 2.2 is developed based on the ePIE update, the same reference-regularization idea can be applied to other reconstruction algorithms such as the rPIE, mPIE [29] or LSQML [30] algorithm by adding the reference prior term to the probe update step. In the present tests, replacing the ePIE probe update with the rPIE probe update with β < 1 did not bring additional benefit to the reconstruction quality. Therefore, the current analysis is restricted to the ePIE method modification.

The central idea of the proposed method is to transfer the reconstructed probe between the high-overlap region and low-overlap region reconstructions, thereby alleviating the lack of constraint in sparse-scan reconstruction. This strategy does not rely on the periodicity or structural similarity of the sample. The method was further evaluated on a non-periodic biological sample whose structure varies markedly across the FOV (Supplementary Figure S6), demonstrating the effectiveness of the method for general samples.

TV- and sparsity-based methods introduce regularization into the object reconstruction, and their performance depends sensitively on the selected regularization strength. In contrast, the proposed method applies the reference-guided constraint to the probe update in sparse area. The sensitivity analysis in Supplementary Figure S7 shows that, once the reference-guiding term is activated, the reconstruction quality is insensitive to the specific value of the regularization strength γ over the tested range. This insensitivity demonstrates a clear advantage of our method over TV- and sparsity-based object regularization in parameter tuning, and it justifies the choice of γ = 1.0 in the above reconstructions. From a perspective of residual-driven reconstruction, the regularization term in the local loss function initially imposes a strong $l_{2}$ constraint that outweighs the far-field residual; however, as the reconstruction stabilizes, the probe is anchored near the reference, allowing the update process to return to a residual-dominant status.

The proposed hybrid-overlap scanning strategy should be interpreted as an acquisition–reconstruction trade-off rather than a purely post-processing strategy. In the experimental result presented here, the 2.0 µm step scan dataset contains 625 diffraction patterns. For comparison, a full-FOV high-overlap scan with a 1.0 µm step size would produce 1225 patterns, approximately twice the number used in the experiment. In addition, if the partitioning treatment method was excluded and only one high-overlap region was used, the total pattern number would be 429, corresponding to 35.0% of the full high-overlap acquisition. The current experimental reconstructions yield sub-20 nm resolutions, suggesting that the proposed acquisition method is favorable for large-FOV ptychography.

With a fixed exposure time per-position, reducing the number of diffraction patterns directly results in shorter scan time and lower accumulated exposure dose. This consideration is particularly important for ptychographic tomography, where the number of ptychography diffraction patterns is multiplied by the number of projection angles and the accumulated radiation dose can become severely high. At the same time, full-FOV coverage is essential for tomographic reconstruction. The hybrid-scan ptychography proposed here therefore provides a practical way to retain full-FOV coverage with high resolutions while significantly reducing the pattern number relative to a pure high-overlap acquisition.

## 5. Conclusions

In summary, we proposed a new sparse-scan ptychography method that combines a hybrid-overlap scanning strategy and a reference-guided ePIE algorithm. In this method, a small high-overlap scanning region is used to recover a high-quality probe, which is utilized in the guiding term of the reference-guided ePIE for the large low-overlap region reconstruction. The simulations with FZP-type and KB-type probes indicate that the proposed method can greatly alleviate low-overlap reconstruction degradation. The multi-Siemens-star experiment demonstrates that this method can be implemented in a realistic soft-X-ray beamline with a sparse-scan regime. The results indicate that the acquisition efficiency of the new method is at least two times better than a conventional method without significant resolution reduction.

As an acquisition–reconstruction compromise strategy, the reference-guided reconstruction idea of the strategy can be applied to other ptychography methods by adding the reference-regularization term. This strategy has great potential in application scenarios such as ptychographic CT, where scan time or radiation dose limits restrict the feasibility of high-density acquisition.

## Figures and Tables

**Table 1:** Parameters in both the simulation and experiment.

Parameters	FZP Simulation	KB-Type Simulation	Experiment
Photon energy	774 eV	774 eV	774 eV
Whole FOV	30 µm × 30 µm	30 µm × 30 µm	36 µm × 36 µm
Probe size	~4 μm	~4 μm	~4 μm
Probe shape	Circular	Quasi-square	Annular
High-overlap step size	1 μm	1 μm	1 μm
High-overlap scan range	7 μm × 7 μm	7 μm × 7 μm	7 μm × 7 μm
Low-overlap step size	2.8, 2.4, 2.0, 1.6, 1.2 μm	3.8, 3.2, 2.6, 2.0, 1.4 μm	2.0, 1.9, 1.8, 1.7 μm
Linear overlap ratios	30, 40, 50, 60, 70%	5, 20, 35, 50, 65%	50, 52.5, 55, 57.5%

## Data Availability

Data will be made available upon reasonable request.

## Supplementary Materials

The following supporting information can be downloaded at https://www.mdpi.com/article/10.3390/s26165168/s1, Figure S1: Simulation design for FZP and KB-type probes; Figure S2: FZP simulation results for low-overlap jittered raster scans; Figure S3: KB-type probe simulation results for low-overlap jittered raster scans; Figure S4: Radial momentum-transfer distribution of the 2.0 μm-step experimental dataset; Figure S5: FRC and MSE analyses of the four experimental reconstructions for the low-overlap step sizes of 2.0, 1.9, 1.8, and 1.7 μm, respectively; Figure S6: Experimental reconstruction results of a biological specimen with structural heterogeneity; Figure S7: Simulation results under four different reference-guiding strengths for seven different SNR levels of Poisson noise.
