# Supplementary material for: Sparse-Scan Ptychography with Hybrid-Overlap Scanning and Reference-Guided Probe Updates
Source: Sensors (Basel). 2026 Aug 14;26(16):5168. doi: 10.3390/s26165168 (PMC13517391; doi:10.3390/s26165168)
Supplement: Supplementary file 1 [file sensors-26-05168-s001.zip › sensors-4425954-supplementary.pdf]

sensors

MDPI

S1. Simulation on jittered raster grids for low-overlap scans

To further validate the robustness of the proposed hybrid-scan ptychography method, we performed additional simulations using jittered raster trajectories for low-overlap scans. Both FZP-type and KB-type probe models were considered here. For each probe model, the hybrid-overlap scan combines a low-overlap jittered raster scan over the entire FOV with a local high-overlap scan in the central area.

The jittered raster grids for low-overlap scan were generated by randomly offsetting each raster position within ±0.1 times of the nominal low-overlap step size in both lateral directions. Other conditions, such as noise level, were the same as that used in the simulations described in the main text.

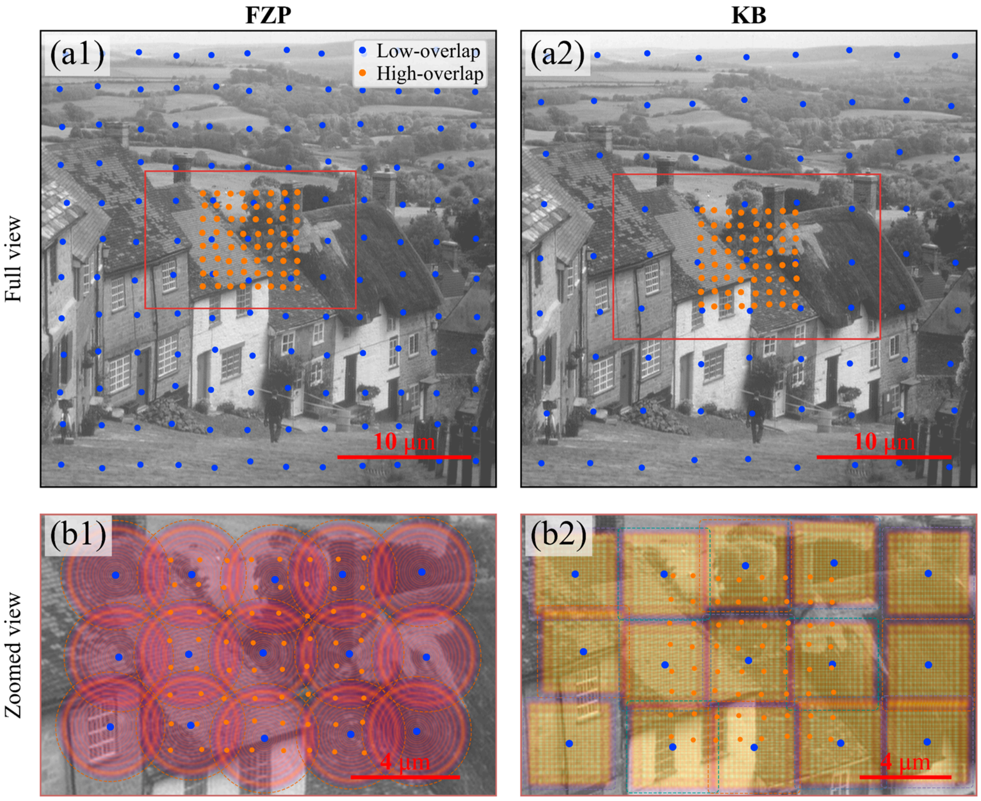

Figure S1. Simulation design for FZP and KB-type probes. (a1) Scan positions in the FZP simulation for a low-overlap step size of 2.8 μm; the blue and orange points denote low-overlap and high-overlap positions, respectively. (a2) Scan positions in the KB-type probe simulation for a low-overlap step size of 3.8 μm; the blue and orange points denote low-overlap and high-overlap positions, respectively. (b1, b2) are the enlarged red-boxed regions of (a1, a2), respectively, showing the footprints of the illumination spots.

Fig. S1 illustrates the simulation geometries of FZP and KB-type models at the largest low-overlap step sizes of 2.8 μm and 3.8 μm, respectively. Figs. S1(a1, a2) show the full FOVs covered by sparse scans, and Figs. S1(b1, b2) show the enlarged views of the central area to demonstrate the illumination status. Under a strict intensity threshold, the jittered raster scan results in scattered blind regions on the object, which are only covered by peripheral low-intensity tails of the illumination. The reconstructed amplitude and phase images of the FOV for the hybrid-overlap and low-overlap-only schemes are presented in Figs. S2 and S3, for the FZP and KB-type probe models, respectively.

 https://doi.org/10.3390/s26165168

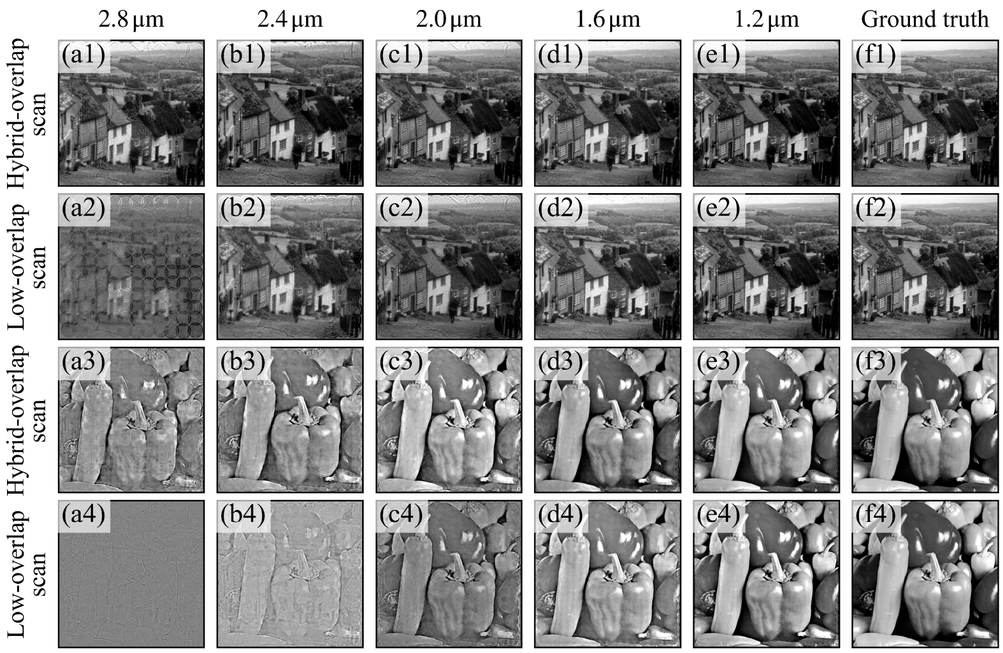

Figure S2. FZP simulation results for low-overlap jittered raster scans. Columns (a-e) are reconstruction results for low-overlap step sizes of 2.8, 2.4, 2.0, 1.6, and 1.2 µm, respectively. Column (f) is the ground truth. Rows (1-4) correspond to the hybrid-overlap amplitudes, low-overlap-only amplitudes, hybrid-overlap phase shifts, and low-overlap-only phase shifts, respectively.

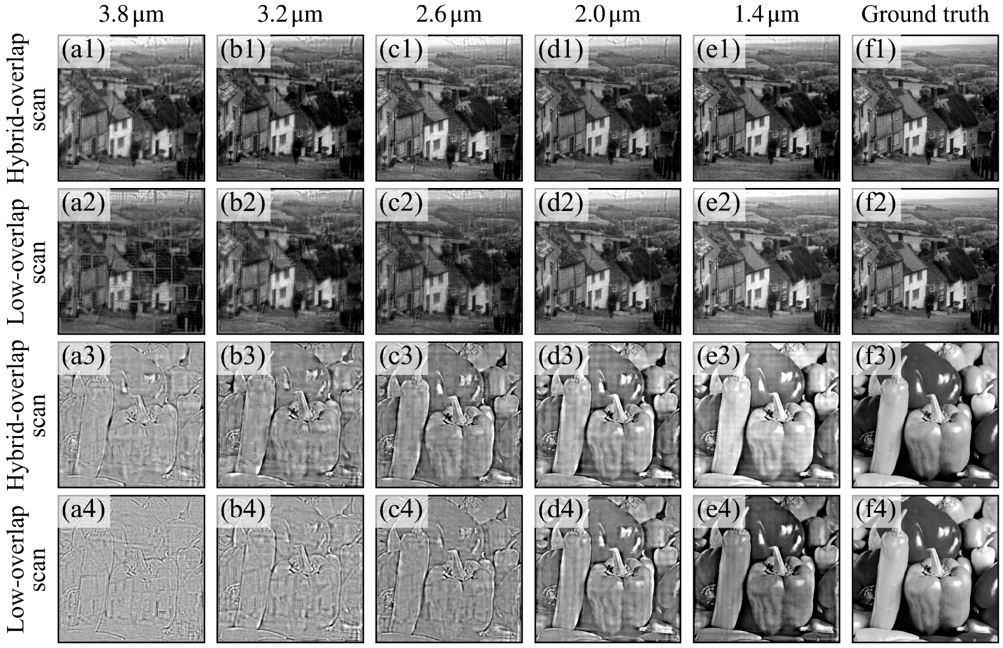

Figure S3. KB-type probe simulation results for low-overlap jittered raster scans. Columns (a-e) are reconstruction results for low-overlap step sizes of 3.8, 3.2, 2.6, 2.0, and 1.4 µm, respectively; column (f) is the ground truth. Rows (1-4) correspond to the hybrid-overlap amplitudes, low-overlap-only amplitudes, hybrid-overlap phases, and low-overlap-only phases, respectively.

Figs. S2-S3 show the same overall trend as the results for low-overlap regular raster scans presented in the main text. When the scan step size is increased, the reconstruction quality of low-overlap-only scans rapidly decreases for both the FZP-type and KB-type probes. In contrast, the hybrid-overlap scan substantially improves the reconstruction quality at large step sizes by introducing a local high-overlap region, which provides additional ptychographic constraints on the sparse full-FOV scan.

https://doi.org/10.3390/s26165168

The results at the intermediate step sizes also illustrate the distinction between jittered and regular raster scans. When a low-overlap regular raster scan could support reasonably stable reconstructions, introducing position jitters can mitigate periodic grid artifacts. However, a regular raster scan yields more uniform spatial coverage across the FOV, avoiding the emergence of blind regions on the object where no illumination spot passes by. Thus the jittered and regular raster scan settings reflect a trade-off between artifact suppression and coverage uniformity. Importantly, in both cases, the hybrid-overlap strategy consistently improves reconstruction robustness, confirming its effectiveness regardless of specific trajectories.

S2. Momentum-transfer range of the experimental diffraction patterns

To characterize the radial momentum-transfer range recorded in the experiment, we analyzed the dataset acquired with a 2.0 μm scanning step. The diffraction intensities were averaged over all scan positions, and the resulting mean diffraction pattern and its radial intensity profile are shown in Fig. S4. The azimuthally averaged diffraction intensity profile was fitted over 18–50 μm^{-1} using the equation of $I(f) = B(f/f_0)^{-n} + C$, where the power-law term describes the decay of the diffraction signal, C denotes the residual intensity floor, and f_0 is a fixed reference spatial frequency.

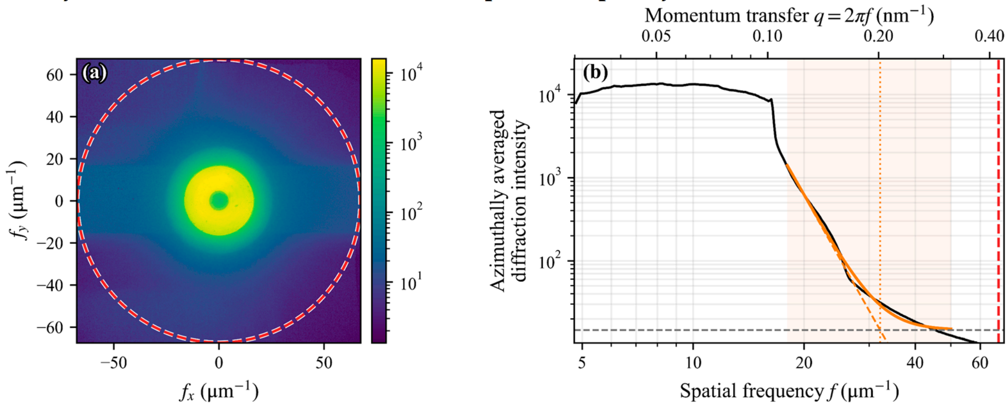

Figure S4. Radial momentum-transfer distribution of the 2.0 μm-step experimental dataset. (a) Logarithmically scaled mean diffraction intensity; the dashed circle denotes the boundary of complete azimuthal coverage. (b) Azimuthally averaged diffraction intensity profile and its power-law-plus-constant fitting plotted on log–log axes. The dashed orange and gray lines show the two fitted components, while the orange and red vertical lines indicate their crossover position (critical spatial frequency) and the detector-coverage boundary, respectively.

As indicated in Fig. S4(a), with an X-ray photon energy of 774 eV, the detector provided a complete azimuthal coverage of momentum transfer up to $0.423\ \mathrm{nm}^{-1}$. From the fitted profile in Fig. S4(b), the crossover position (critical spatial frequency) between the power-law decrease component and the residual intensity floor was estimated to be approximately $0.20\ \mathrm{nm}^{-1}$, defining the upper boundary of the effective momentum-transfer range.

S3. Quantitative evaluation of experimental results for varying step sizes

To quantitatively evaluate the experimental reconstruction results for the four different low-overlap step sizes presented in the main text (Fig. 10), we provide Fourier Ring Correlation (FRC) and Mean Squared Error (MSE) analyses here.

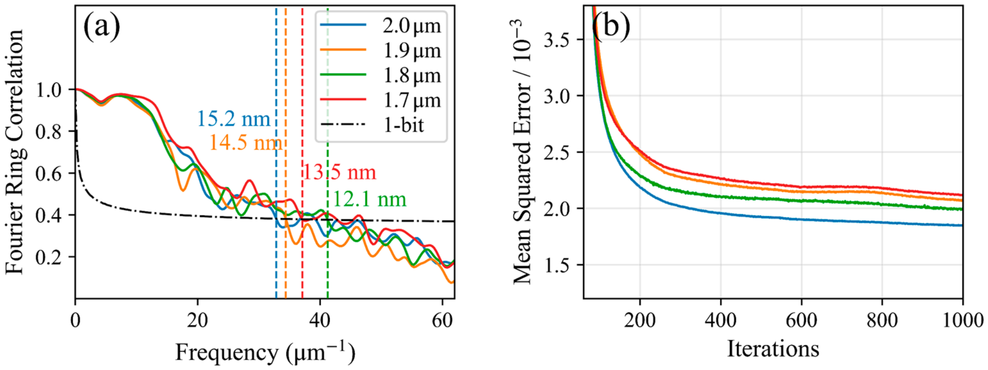

Figure S5. FRC and MSE analyses of the four experimental reconstructions for the low-overlap step sizes of 2.0, 1.9, 1.8, and 1.7 μm, respectively. (a) FRC curves with the 1-bit threshold shown together. (b) Normalized far-field amplitude MSE curves for the four reconstructions. For each step size, the FRC and MSE curves were obtained from one representative reconstruction run.

As shown in Fig. S5(a), based on the 1-bit threshold criterion, the reconstructions achieve spatial resolutions of 15.2 nm, 14.5 nm, 12.1 nm, and 13.5 nm for the step sizes of 2.0 μm, 1.9 μm, 1.8 μm, and 1.7 μm, respectively. Furthermore, the MSE curves shown in Fig. S5(b) demonstrate that the iterative reconstruction process converges stably across all tested step sizes. It should be noted that the achieved resolution does not strictly depend on the step size; rather, it depends more significantly on the stability of the scanning process and a high SNR.

S4. Experimental validation on a biological specimen

To examine whether the proposed method depends on structural similarity between the high- and low-overlap regions, we reanalyzed an existing experimental ptychography dataset acquired from a cell specimen (a kidney cell from an African green monkey) with nonperiodic features. The full dataset was reconstructed as a baseline, and the same dataset was then used to form (i) a hybrid-overlap dataset containing a low-overlap scan across the entire FOV and a local high-overlap scan and (ii) a low-overlap-only dataset containing the same low-overlap scan positions. The reference-guided and sparse-data reconstructions therefore share the identical sparse full-FOV measurements, while the former additionally uses the localized high-overlap data to obtain the reference probe constraint. For the hybrid-overlap dataset, the low-overlap scan and high-overlap scan shared some scan positions.

https://doi.org/10.3390/s26165168

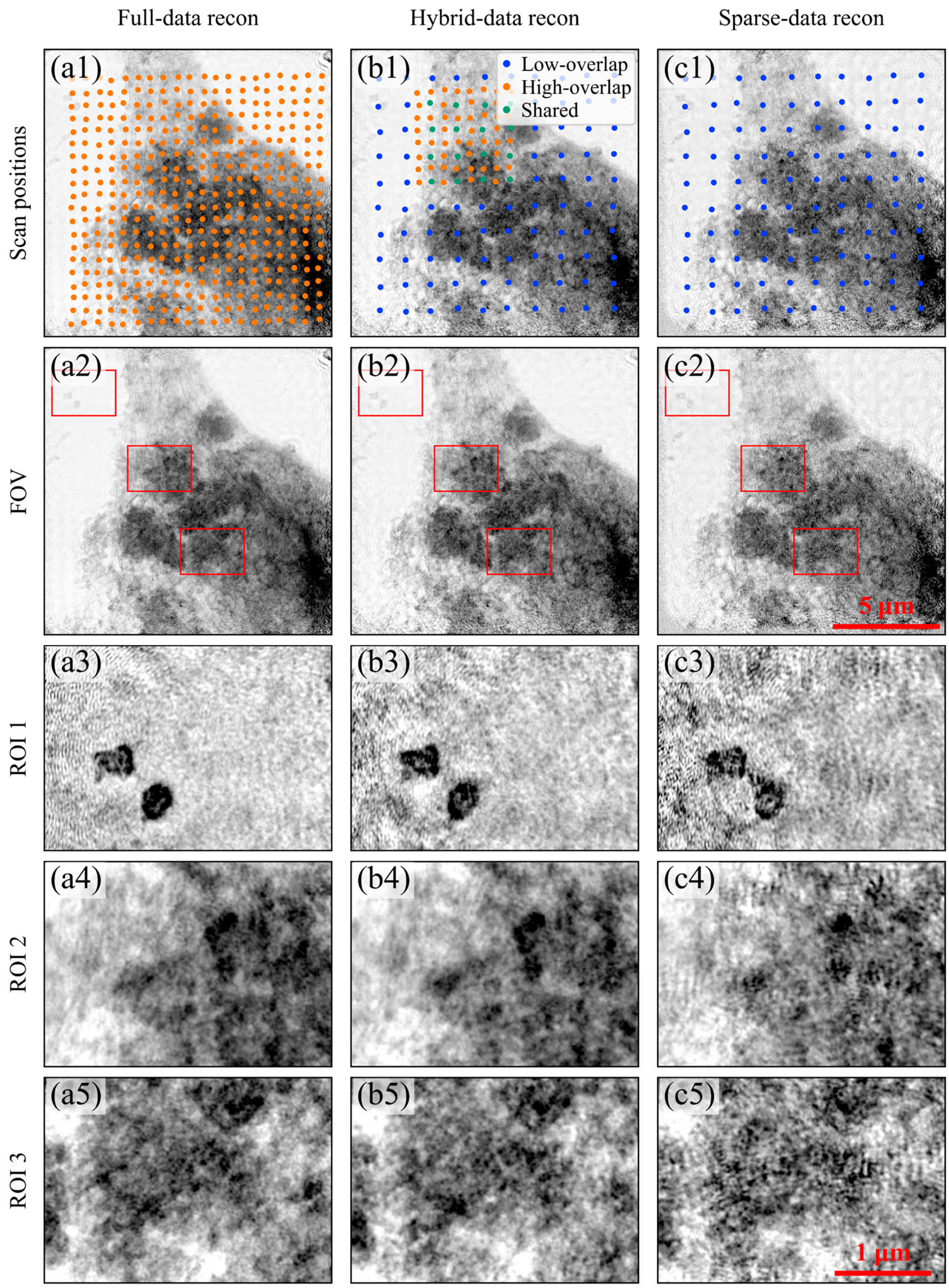

Figure S6. Experimental reconstruction results of a biological specimen with structural heterogeneity. Reconstructed object amplitudes are shown here. Columns (a-c) show the full-data, hybrid-data, and sparse-data reconstruction results, respectively. Row (1) shows the scan positions for the three reconstructions, row (2) shows the reconstructed full FOVs for the three cases, and rows (3-5) show enlarged views of ROIs 1-3, respectively. The blue, orange, and green points in the first row of panels denote the low-overlap, high-overlap, and shared scan positions, respectively.

Fig. S6 compares the three reconstruction results for the full FOV and three representative regions of interest (ROIs). Compared to the sparse-data reconstruction, the hybrid-data reconstruction better agrees with the full-data reconstruction across the FOV. This improvement is also evident in ROI 3, which lies entirely outside the local high-overlap region and contains a sample structure clearly different from that within the high-overlap region. For hybrid-data reconstruction, the high- and low-overlap object estimates remain separate, and only the reference probe is used to guide the low-overlap region

reconstruction. These results demonstrate that the proposed method does not require similar sample structures between the high-overlap and low-overlap regions.

S5. Sensitivity to the reference-guiding strength parameter γ

To assess the numerical stability and robustness of the proposed reconstruction strategy, we investigated the sensitivity of the reconstruction quality to the reference-guiding strength parameter γ by a series of numerical simulations (Fig. S7). These tests were performed under extreme parameter conditions to probe the limits of the algorithm. Specifically, the warmup iteration was omitted (N_{warm}=0), requiring the algorithm to establish stability through the reference-guided updates from the very first iterations. Furthermore, the simulations were pushed to an extremely low signal-to-noise ratio of SNR = 1 to explore the limit cases.

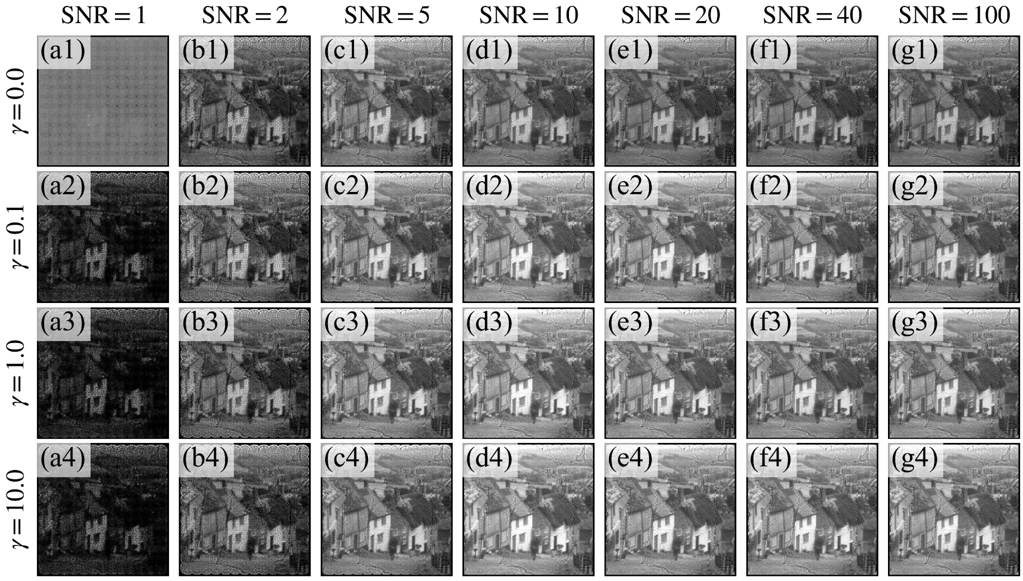

Figure S7. Simulation results under four different reference-guiding strengths for seven different SNR levels of Poisson noise. Each column corresponds to a specific SNR, ranging from 1 to 100. Each row corresponds to a strength parameter value. Only reconstructed amplitudes are shown here.

The comparison across various noise levels demonstrates that any activation of the reference-guiding strength parameter (γ>0) yields superior results compared to the standard ePIE approach (γ=0). Although the transition from γ=0 to γ>0 is essential for maintaining structural integrity in high-noise regimes, further fine-tuning of reference-guiding strength γ (e.g., from 0.1 to 10.0) does not show observable improvement in reconstruction quality.
